# Coastal Image Classification and Pattern Recognition: Tairua Beach, New Zealand

**DOI:** 10.3390/s21217352

**Published:** 2021-11-05

**Authors:** Bo Liu, Bin Yang, Sina Masoud-Ansari, Huina Wang, Mark Gahegan

**Affiliations:** 1 School of Software Engineering, Faculty of Information Technology, Beijing University of Technology, Beijing 100124, China; yangbin@emails.bjut.edu.cn (B.Y.); whn@emails.bjut.edu.cn (H.W.); 2 School of Fundamental Sciences, Massey University, Palmerston North 4472, New Zealand; 3 Centre for e-Research, The University of Auckland, Auckland 1010, New Zealand; s.ansari@auckland.ac.nz (S.M.-A.); m.gahegan@auckland.ac.nz (M.G.); 4 School of Computer Science, The University of Auckland, Auckland 1010, New Zealand

**Keywords:** coastal image, convolutional neural networks, beach state classification, pattern recognition

## Abstract

The study of coastal processes is critical for the protection and development of beach amenities, infrastructure, and properties. Many studies of beach evolution rely on data collected using remote sensing and show that beach evolution can be characterized by a finite number of “beach states”. However, due to practical constraints, long-term data displaying all beach states are rare. Additionally, when the dataset is available, the accuracy of the classification is not entirely objective since it depends on the operator. To address this problem, we collected hourly coastal images and corresponding tidal data for more than 20 years (November 1998–August 2019). We classified the images into eight categories according to the classic beach state classification, defined as (1) reflective, (2) incident scaled bar, (3) non-rhythmic, attached bar, (4) attached rhythmic bar, (5) offshore rhythmic bar, (6) non-rhythmic, 3-D bar, (7) infragravity scaled 2-D bar, (8) dissipative. We developed a classification model based on convolutional neural networks (CNN). After image pre-processing with data enhancement, we compared different CNN models. The improved ResNext obtained the best and most stable classification with *F1*-score of 90.41% and good generalization ability. The classification results of the whole dataset were transformed into time series data. MDLats algorithms were used to find frequent temporal patterns in morphology changes. Combining the pattern of coastal morphology change and the corresponding tidal data, we also analyzed the characteristics of beach morphology and the changes in morphodynamic states.

## 1. Introduction

Changes in beach morphology are the result of the interaction between ocean dynamic factors (waves and tides) and topographic dynamic factors (sediment and topography). Understanding beach response to waves and currents is necessary to improve predictions of beach change.

The classification of beach morphology, the so-called “beach state”, is one of the steps undertaken by researchers to characterize typical beach behavior and to identify transitions between states. At present, most researchers base beach classification on expert judgment of available images or using statistics and multivariate analysis of key environmental factors (such as wave height, wave period, sand particle size, beach slope, tidal range, and bay state). Existing classification models initially focused on wave-dominated microtidal beaches on open coasts [1,2,3], but were subsequently developed for all types of tidal range [4,5] and different beach types [6,7]. Beach classification models are usually based on a large number of temporal and spatial observations and are suitable for environments where observational data are routinely collected. In other words, a beach state classification model requires datasets that are dense in time (many observations over the long-term are needed) and extensive in space (a large portion of the beach needs to be available for analysis). The dataset should also include detailed morphological, sedimentological, and hydrodynamic information for the beach of interest. However, due to the environmental and technical constraints, there are few long-term datasets that are suitable for classification. Lippmann and Holman [3] collected a dataset spanning 2 years, which consists of daily time exposure images of incident wave breaking on an open coast. Beach states are usually classified into eight categories based on the general understanding of beach morphodynamics, and six categories could be observed for the beach analyzed. In addition, the classification of the images was entirely dependent on experts, which implies a heavy workload and an element of subjective uncertainty. To address these aspects, we used a long-term dataset of images of an embayed beach in New Zealand. The images cover almost the entire length of the beach and were collected hourly for over 20 years (Nov. 1998–Aug. 2019). The beach types in the dataset range from reflective to dissipative and only one of the states described in [3] is not observed.

Statistical analysis [8,9] and cluster analysis [10,11], as technologies for identifying structures in multivariate datasets, were used in the study of beach morphology dynamics and classification. However, the current beach classification models are based on local environmental parameters (a notable exception is discussed in the next section), which limits the applicability and generality of the findings. On the other hand, shore-based surveillance video has the characteristics of non-contact, low cost, and spatio-temporal continuity, so the video imaging and analysis technology based on shore-based surveillance video has been widely used in the study of coastal morphological dynamics. For many years, coastal studies [12] have been using coastal video to measure or track broken waves [13,14,15], estimate wave height and wave period [16,17], segmentation of coastal images [18], and coastline detection [19]. These studies based on coastal video applications show that it is effective to extract the relevant feature information of waves and sandbars using computer vision technology, especially the data involving machine learning algorithms. However, few detailed studies of near-shore wave domain and sandbar detection have inspired us to develop beach state classification using only beach image data.

Beach classification model is a qualitative conceptual model, which identifies different patterns of beach state according to environmental conditions (wave climate, tidal conditions, and beach sediment characteristics). One current beach model needs to design the corresponding classification model according to the environmental parameters (breaking wave height, sediment fall velocity, wave period, surf-scaling parameter, relative tide range, etc.) [20], which leads to the fact that one model can only be used in a certain region or certain environmental conditions that bring some limitations for beach research. The other classification method based on beach morphology does not need to rely too much on environmental parameters, but extracts morphological features (sandbanks, shorelines, wave breaking or crack locations, etc.) from monitoring images by traditional video analysis technology to identify specific beach states [21,22,23]. However, this method of identifying beach state needs to artificially set morphological features, so it is highly subjective. In recent years, deep learning has been widely used in various fields, especially in image classification and recognition, showing obvious advantages. Deep learning methods can learn the input–output mapping in a potentially nonlinear fitting way. When we define the coast image and the corresponding category as input and output, the deep learning model can learn the interesting features of the image in an objective way, and then detect the specific patterns (beach categories) in new coastal images (details are shown in Section 4.2).

**Figure 1:**
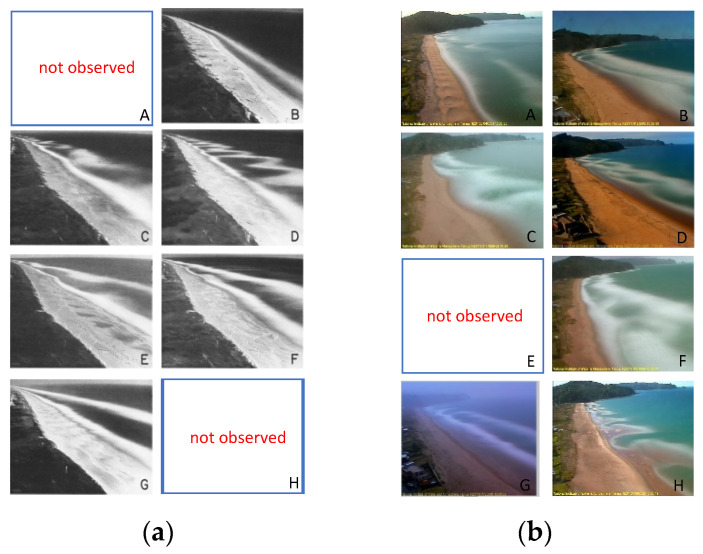
Comparison of Lippmann’s classification (Adapted with permission from Ref. [3]. © 2021 John Wiley and Sons.) (**a**) and the classification in this paper (**b**). There are eight classes: (A) reflective, no offshore bar, and lack of wave breaking; (B) incident scaled bar, little longshore variability, and low tide terrace; (C) non-rhythmic, attached bar, no coherent longshore rhythmic; (D) attached rhythmic bar, rhythmic bar line, and discontinuous trough; (E) offshore rhythmic bar, rhythmic bar line, and continuous trough; (F) non-rhythmic, 3-D bar, longshore variable, non-rhythmic, and continuous trough; (G) infragravity scaled 2-D bar, no longshore variable; (H) dissipative, flat beach slope.

Based on the above analysis, we introduce a deep learning method (convolutional neural network) to automate the process of beach classification. Firstly, we introduced the traditional beach classification [3] where beach states are divided into eight categories defined as (A) reflective, (B) incident scaled bar, (C) non-rhythmic, attached bar, (D) attached rhythmic bar, (E) offshore rhythmic bar, (F) non-rhythmic, 3-D bar, (G) infragravity scaled 2-D bar, (H) dissipative (shown in Figure 1). We did not observe the beach in full the dissipative state but also had several occurrences of a state not observed at the location studied by [3]. This state involves the presence of large rhythmic shoreline undulations under moderate waves. Our state H corresponds to this configuration: rhythmic shoreline sand wave (we reiterate that also [3] did not observe state H). Secondly, the annotated image data which is a small fraction of total dataset is sent into convolutional neural network (classifier) to train a beach classification model. Convolution neural network maps the morphological features of beaches and waves to one of the eight categories, and then the classification model is used to predict the classification of the whole beach dataset after verification by certain evaluation criterion. Notably, in order to make full use of unlabeled images, we introduce a self-training mechanism. The main idea of self-training is to alternate training two models which can be the same or different, namely “teacher” and “student”. In our experiments, “teacher” and “student” models are the same. Initially, the teacher model trains the labeled images, and then predicts the unlabeled images with pseudo labels. These pseudo labels can be soft labels or hard labels converted by using a most confidence criteria. Then, the labeled and unlabeled images are combined, and the student model is trained according to the combined data.

The contributions of this paper are summarized as follows:

(1) We collected a long-term dataset containing video images of a beach in New Zealand for more than 20 years.
(2) We propose an innovative method based on deep learning for automated beach classification which can dynamically extract location and shape information of offshore dam, coastline, and wave of coastal dam trough for classification decision. Moreover, a self-training mechanism is introduced to make full use of unlabeled images which improves the generalization ability of the CNN model. Further, through the strategy, our proposed ResNext achieves the state-of-the-art results (*F1*-score = 0.9014).
(3) A motif discovery algorithm is proposed to recognize beach patterns. After grouping and serializing the data of beach state over the past 20 years which can be discerned by proposed CNN model, the frequent beach patterns and beach state transitions could be recognized every year.

## 2. Previous Work

The classification of beaches has been a widely recognized research topic in the study of coastal morphodynamics. Most of the predictive models of beach state are based on environmental parameters (e.g., dimensionless fall velocity parameter and relative tidal range). Wright and Short [2] synthesized classification studies by Short [24,25] and Wright et al. [26], and posed the foundation of contemporary beach morphodynamics. The classification developed by Wright and Short [2] combined the characteristics of waves and sediments through the dimensionless fall velocity parameter Ω. However, the influence of tide was still not considered. Using the Ω parameter, Masselink and Short [5] proposed the relative tidal range (RTR) to account for the influence of tides on beach morphology in the classification. The study extended the classification to a broader range of beach types. Other studies, e.g., Short [27], added other states or considered geological controls.

Video imaging was also used in nearshore morphodynamics as a technique for quantifying sandbar change. Lippmann and Holman [3] used remote sensing technology (time average imaging of breaking waves in the surf zone), which could quickly estimate the location and longshore variability of the underwater sandbar and of the shoreline. Eight beach states could be defined, and the temporal and spatial variability of sandbank morphology could be described with unprecedented temporal and spatial resolution. Jennings and Shulmeister [28] proposed a method on simple visual classification of gravel beaches in New Zealand, which can be applied globally. Based on the morphological and dynamic differences among the beach types, the beach can be identified and classified objectively by multivariate analysis technology. Kazmer and Taborosi [29] and Almeida et al. [30] used a range of terrestrial laser techniques to generate very high temporal resolution morphological images. The images could be used to deduce beach parameters such as sediment type, surface roughness, surface water content, and vegetation coverage, and then measure morphological change. By using cluster analysis, Scott et al. [31] simplified the beach classification model based on environmental parameters (morphology, sedimentology, and hydrodynamics), defined and identified nine different beach types, and estimated inshore wave conditions combined with observational wave data and numerical modeling. McCarroll et al. [22] proposed a new beach state model for asymmetric, transitional embayed beaches. The model described a pre-storm embayment where beach state changed gradually alongshore, while the post-storm embayment exhibited an extreme alongshore morphological gradient, which was contrary to the existing conceptual model of beach state. Armaroli et al. [32] used Argus technology developing a semi-quantitative beach morphodynamic classification based on the observation of breaking wave patterns. The main limitations of these video-based methods are the time consuming task of producing results and the limited feature extraction by manually designed programs.

Deep learning is a widely used technology to address problems related to computer vision, speech recognition, and natural language processing. As a common deep learning architecture, Convolutional Neural Network (CNN) is inspired by the biological and natural visual cognitive mechanism and has been widely applied in the field of image classification. Common CNN structures include LeNet [33], AlexNet [34], VggNet [35], GoogleNet [36], ResNet [37], DenseNet [38], and so on. The early convolutional neural network has a relatively simple structure, such as the classic LeNet model, which is mainly applied in some relatively simple computer vision applications such as handwritten characters and image classification. Simonyan et al. [35] proposed a new aspect of the network structure and created the stacked structure model. At the same time, the authors pointed out that the depth of the network was the key to improve the model. Szegedy et al. [36] increased the width of the model by multi-channel nonlinear mapping, which greatly improved the expression ability of the model. With the deepening of the depth and width of the model, the phenomenon of gradient obsession and gradient explosion appears in the network model. He et al. [37] proposed skip Connections structure, which made it possible to train a deeper network.

Ellenson et al. [39] used classical CNN models (ResNet50) to classify beach states into five classes according to Wright and Short [2]’s classification scheme. They conducted experiments to evaluate the performance of their CNN model based on imagery datasets from Narrabeen (New South Wales, Australia) and Duck (North Carolina, USA) and showed that CNN had accurately identified key features of the coastal images which distinguished beach states. In Section 4, we will compare our method with theirs in detail.

## 3. Methodology

### 3.1. Coastal Image Classification

#### 3.1.1. Dataset Pre-Processing

Tairua Beach is located on the east coast of the Coromandel Peninsula in the North Island of New Zealand [40]. The beach face at Tairua is generally steep (≈6°) and characterized by medium-coarse sand. The tidal range is between 1.2 and 2 m. Tairua Beach has been monitored since 1998 by a Cam-Era video system installed on the extinct volcano (70.5 m above chart datum) at the south end of the beach. Beach morphology was surveyed over a region encompassing dunes shoreline and surfzone in the cross-shore direction. Additionally, the video system has been collecting 600 time exposure (timex) images over a period of 20 min every hour. Each set of 600 hourly timex images was averaged to remove the effect of random breaking waves on light intensity patterns. When breaking wave happened, hourly timex images showed location and morphology of submerged nearshore sand bars and rip channels [3]. Time-exposure images were also widely exploited in shoreline detection [41], breaking wave height [42], wave period [43], and bathymetry [44]. Therefore, hourly timex images can be used to classify the beach state in this paper.

In our dataset, there are 103,660 images of which 3744 images are labeled. Our dataset has two resolutions: 760 × 570 and 2016 × 1528. In terms of the classic classification proposed by Wright and Short [2], Tairua Beach lacks E types.

In addition, poor quality images were discarded from the dataset. The images (1998–2019) were used to build our classification model while only a subset of the data collected from the years 1999 to 2008 was used for analysis.

The performance of the deep learning algorithm is highly dependent on data. Generally speaking, the higher the quality and quantity of the dataset, the better the training of the model. However, some problems exist in the dataset: (1) complex lighting conditions: light conditions change frequently as a result of cloud coverage and sun position with respect to the camera; (2) complex environmental conditions: rain or dust blocked out the lens, making the picture noisy; (3) camera shaking: it can vary the angle of view during image collection and result in blurred image ghosting. Therefore, in order to reduce the impact of adverse factors on the algorithm, data cleaning and data pre-processing were conducted.

Data cleaning aims to remove the images that are too noisy for a variety of reasons. We checked for beach type images that are clearly misclassified. Various image similarity algorithms are used to remove similar images.

In data pre-processing, we used data enhancement methods like random crop and color transformation. On the one hand, more training samples could be generated (to a certain extent, the problem of sample balance could be solved), the influence of size, translation, and color on the model are reduced, and the classification accuracy and generalization ability of the model are further improved. On the other hand, image enhancement improves the generalization ability and robustness of the model by increasing the data volume of the training set. Each input image is normalized through mean RGB-channel subtraction.

#### 3.1.2. Model Design

Convolution neural network, as a common and crucial network of deep learning, achieves the state-of-the-art performance in the field of image classification. The beach classification model based on CNN contains the convolutional layer and the fully connected layer. The algorithm consists of a convolution layer, pooling layer, and activation function layer to map the original data to the hidden layer feature space, a process similar to extracting image features. The fully connected layer, which is similar to the classifier, maps the feature representation learned by the former to the sample label space (Category label). In the training process of the deep learning model, an error will be obtained when the predicted result of the network is compared with the truth. This error will be transmitted (back propagation) in each layer of the model, and the representation of each layer will be adjusted according to this error. The process will not end until the model converges or achieves the expected effect, which is end-to-end.

**Figure 2:**
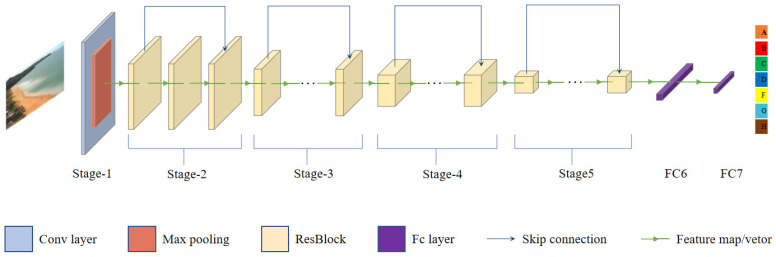
Model structure.

In our research, we used ResNet50 and ResNext50 networks to classify the coastal dataset. The architecture is shown in Figure 2. In contrast to VGG and Inception network structures, ResNet addresses the problem of training set accuracy decreasing as the network deepens. The ResNext could improve the accuracy without increasing the complexity of parameters, while further reducing the number of hyperparameters.

At the input stem stage of ResNet, a 7 × 7 convolution kernel performs feature extraction, which reduces the length and width of the input image to 1/2 of the original. After, the max pooling layer further reduces the resolution of the image and extracts features in a series of stages. Stages 2–5 use repeated residual blocks to extract features. In this part of the operation, the basic idea is to expand the input feature map to double the number of channels and reduce the length and width to 1/2. Specifically, each stage is composed of a down-sampling block and two residual blocks. The initial convolution step size of the down-sampling block is set to 2. In this way, the length and width can be reduced. In the residual block, by setting the parameters of the convolution, the input and output feature maps of the residual block could be set to the same size, so that they can be added together. The problem of vanishing gradient and exploding gradient of deep networks could be avoided with this method.

In addition, ResNext has a simple structure, which is similar to ResNet, easy to understand and powerful enough for the problem. It adopts VGG/ResNets’ strategy of repeating layers, while exploiting the split–transform–merge strategy. On the 1000-class ImageNet classification task [45], it achieves better results than the ResNet/Inception/Inception ResNet series.

**Figure 3:**
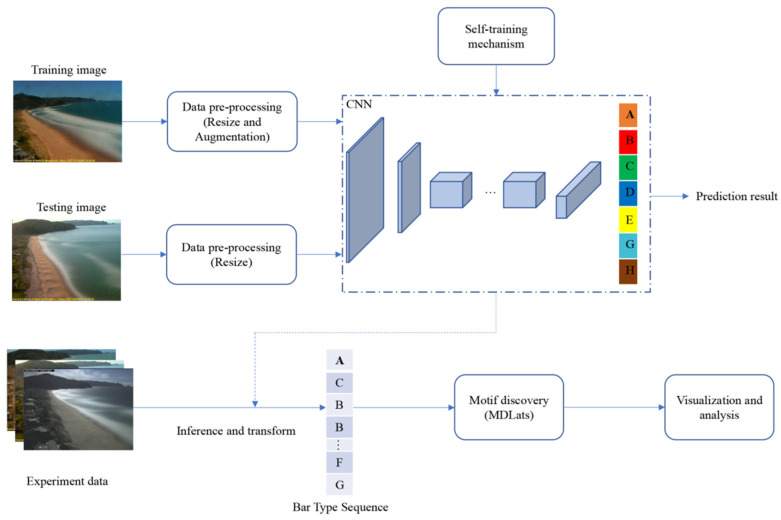
Overview of the proposed method.

Figure 3 shows one epoch of the iterative procedure of training and testing. During the training procedure, the size of the input image is firstly cropped to 256 × 256 and handled by augmentation method. Then, the convolutional layer maps the training data to the hidden layer feature space, while the fully connected layer maps the learned distributed feature representation to the sample marker space (confidence vector of length 7). Finally, the maximum confidence corresponding with the category is the predicting result which is compared with corresponding label to compute loss. Additionally, loss function and optimizer are used to minimize the loss until the convergence of the model. During the testing procedure, the input image is resized to 640 × 640 to ensure consistency with the image size in the training stage. Then, the processed data is fed to the trained model to predict the beach state which is used to evaluate the performance of the model.

#### 3.1.3. Self-Training Strategy

In supervised learning, the model needs a large number of labeled datasets for fitting, and data and manpower costs are usually very high. Generally speaking, the larger the number of training samples, the higher the classification accuracy of the trained classifier. In our dataset, only 3.6% of all images are labeled. Therefore, we propose a self-training strategy, with plenty of unlabeled data to expand a small amount of labeled data. This will improve the accuracy of the model, reduce the model overfitting, and improve the generalization ability of the model.

The detailed algorithm of self-training mechanism is illustrated in Algorithm 1. It is easy to understand the algorithm by dividing it into three terms. The first term (line 3) is training a teacher model θ on the labeled dataset $D^{l}$ with the standard cross entropy loss. The second term (line 5–8) is generating a combined dataset $D^{c}$ containing target soft (a continuous distribution) or hard (a one-hot distribution) labels, which is predicted by the teacher model $\theta^{t}$. The last term (line 9–10) is a student model with noise (data argument) that can force the student to ensure prediction consistency across augmented versions of an image and prevent over-confident predictions of target pseudo-labels on the combined dataset $D^{c}$. In this way, the student model is able to revisit the previous knowledge and avoid catastrophic forgetting of previously learned categories. Finally, by iterating the process (line 5–10) until stopping the process using a criterion such as convergence and maximum iterations, we can get the model with the best performance.

In addition, because the labeled and unlabeled image datasets have the same source and distribution, the teacher and student model can also be the same which greatly reduced training time.
**Algorithm 1.** Self-training.
1: **Input:** Given labeled images $D^{l}=\{(x_{1},y_{1}),(x_{2},y_{2}),...,(x_{n},y_{n})\}$ and unlabeled images $D^{ul}=\{{\bar{x}}_{{}_{1}},{\bar{x}}_{{}_{2}},...,{\bar{x}}_{m}\}$ 2: **Output:** Joint model θ 3: Train a standard teacher classifier $\theta^{t}$ on $D^{l}$
$l(y_{i},f(x_{i},\theta^{t}))$ 4:     **While** stopping criteria not met **do** 5:           Use $\theta^{t}$ to predict class label ${\bar{y}}_{i}$ of $D^{ul}$ 6:           Select confidence sample $D_{conf}=\text{\{}x_{conf},y_{conf}\text{\}}=\{({\bar{x}}_{1},{\bar{y}}_{1}),({\bar{x}}_{2},{\bar{y}}_{2}),...,({\bar{x}}_{i},{\bar{y}}_{i})\text{\}}$ 7:           Remove selected unlabeled data $D^{ul}<-D^{ul}-x_{conf}$ 8:           Combine newly labeled data $D^{c}<-D^{l}\cup D_{conf}$ 9:           Train student model $\theta^{s}$ with data argument(noisy) on $D^{c}$ 10:         Generate new teacher model $\theta^{t}<-\theta^{s}$ 11:    **end while**

### 3.2. Motif Discovery

Different from traditional methods of statistical analysis to study the classification results, we try to use a motif discovery algorithm to further study patterns in state transition.

**Figure 4:**
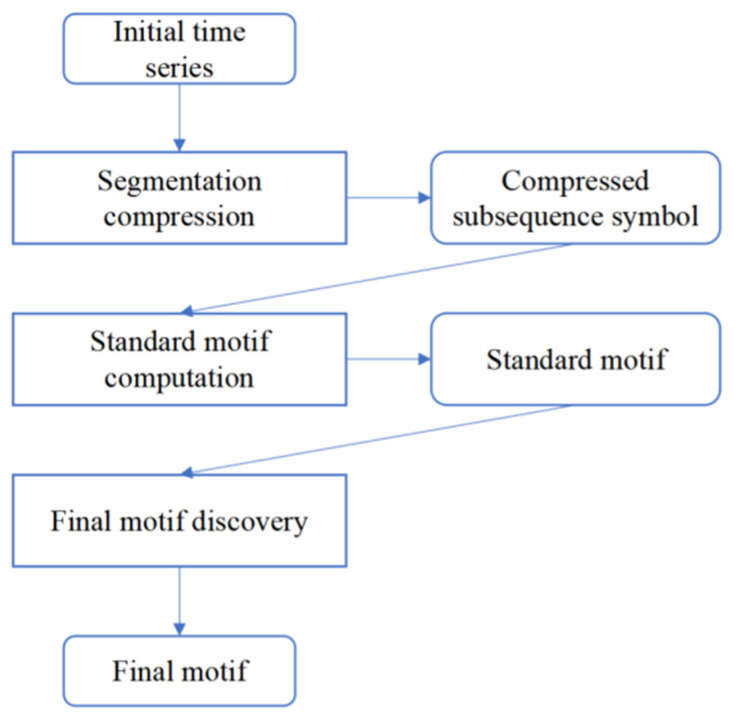
Main steps of MDLats.

Motif discovery is a means of analyzing time series data which can reveal the temporal behavior of the underlying mechanism producing the data. Time series motifs, which are similar subsequences or frequently occurring patterns, have significant meanings for researchers in many domains such as healthcare [46], geonomy [47], and anomaly detection [48]. After establishing the classification model, we can use the whole dataset to extract sequences of states. Since the images are captured in succession, they can be easily transformed into a time sequence. Thus, we use the MDLats [46] algorithm (shown in Figure 4) to look for subsequences which represent a type sequence of beaches that often appears within a certain part of the time series or patterns that occur frequently.

Specifically, a time sequence of beach states firstly split by day were used to generate the initial time series. Secondly, we used a segmentation compression module to eliminate some redundant subsequence. Then, the compressed subsequence symbol was sent to a standard motif computation module to compute standard motif which can reduce the majority of redundant computation. Next, the final motif discovery module compared the extracted subsequence with standard motifs to generate a set of final motifs that have similar motif types. Finally, we used the final motif for further analysis (details shown in Section 4.2).

## 4. Results

### 4.1. Experiments on Coastal Image Classification

#### 4.1.1. Experiment Setting

To verify the training strategies introduced above and our model ability, we designed five folds cross-validations such that all the datasets are divided into five folds and one fold is used as the test dataset for each experiment. Each network architecture is trained with identical optimization schemes. We followed standard practices and performed data augmentation with random cropping using scale and aspect ratio to a size of 256 × 256 pixels and some image enhancement methods such as contrast, solarize and scaling, and then we used Z-Score normalization to speed up model training and improve model accuracy. Optimization was performed using synchronous SGD with momentum 0.9 and a minibatch size of 20. The initial learning rate was set to 0.001, and divided by 10 for three times using the schedule in Xie et al. [49].

For a multi-class classification problem, we used accuracy, precision, recall, and *F1* score to evaluate our algorithm following [50,51]. For each category, a description of these measures is presented below:

(1) $$\text{Accuracy}=\frac{TP+TN}{TP+FN+FP+TN}$$

(2) $$Precision=\frac{TP}{TP+FP}$$

(3) $$Recall=\frac{TP}{TP+FN}$$

(4) $$F1score=2\frac{Precision*Recall}{Precision+Recall}$$

where *TP* is the number of correctly classified positive examples; *FP* is the number of incorrectly classified as positive example; *FN* is the number of incorrectly classified as negative; *TN* is the number of correctly classified negative examples.

All indices range between 0 and 1 with a higher value indicating better performance.

#### 4.1.2. Classification Results

Image enhancement. We used the original images and the images after image enhancement to train two models. The results are shown in Table 1, and it can be seen that: Comparing the accuracy of ResNet50 and ResNext50 with different data processing methods, it is clear that the accuracy obtained using Resnext50 is higher than ResNet50. In particular, the accuracy of ResNext50 is higher than that of ResNet50 by 1.2% and 0.9%. This is because ResNext not only adopts duplicate layer strategy compared to ResNet, but also increases the number of paths which can capture more contextual information. The advantage is to improve the accuracy through a wider or deeper network under the premise of ensuring flops and parameter quantity. Compared with the same network before and after using image enhancement, we can find that the accuracy of the two methods is improved by 2.7% and 2.3%, respectively, which proves the usefulness of image enhancement. The accuracy of the two networks is more than 90%, which proves that our method can accomplish the classification task well.

#### 4.1.3. Self-Training

We designed a self-training mechanism to utilize the unlabeled coastal images and improve predictive ability. The *F1*-score is a measure of multiple classification task, which is a harmonized average of accuracy and recall rates. The performance of self-training is compared for no self-training mechanism and using a self-training mechanism.

As shown in Table 2, ST is the abbreviation of self-training. The average *F1*-score increased by 1.6 percentage points overall, but the *F1*-score of each category improved except for a slight decrease in type D. In each round of self-training, different proportions of unlabeled images and corresponding pseudo-labels were added to the training dataset to assist the training, help categorize, and smooth the prediction indexes of each category. The best result in each category is marked in bold.

#### 4.1.4. Comparison with Other Methods

We analyzed the *F1*-score for the seven beach states using two different classification networks: ResNet50 (used by Ellenson et al. [39]) and ResNext50 (as seen in Table 3).

In the *F1*-score of the two networks, the score of type A and type G is obviously higher than the others. We consider that type A is calm conditions and type G presents a thick straight line of wave breaking whose shape is easy to distinguish, while other types are more complex and have similar appearance, which makes it hard to classify.

Due to the strong feature extraction capabilities of images, ResNext50 has a higher *F1*-score than ResNet50 in all categories except type D. However, types B and H of both models have a lower score. This is because the types B and H are plesiomorphic to some degree and it is likely that there are classification errors in the labels of the dataset.

In addition, we analyzed the Precision, *F1*-Score, Recall, and Referring Time of different classification networks. All models use the same settings. They are pre-trained on ImageNet using the SGD optimizer and use a common loss function of cross entropy. Although self-training can improve model performance, it is time-consuming. Therefore, we did not use the self-train mechanism in this experiment.

As shown in Table 4, it can be clearly seen that ResNext50 achieves the best performance both in Recall and *F1*-score (0.9308 and 0.9014, respectively). VGG16 achieves the lowest scores, which indicates that VGG16 lacks robustness and performs quite poorly for this task. VGG16 and DenseNet121 achieved similar *F1*-Score, but the recall of ResNet50 was lower than that of ResNet50 and ranked as the second of all methods, while its referring time is much faster than Densenet121 (nearly half time of DenseNet121).

#### 4.1.5. Saliency Maps

**Figure 5:**
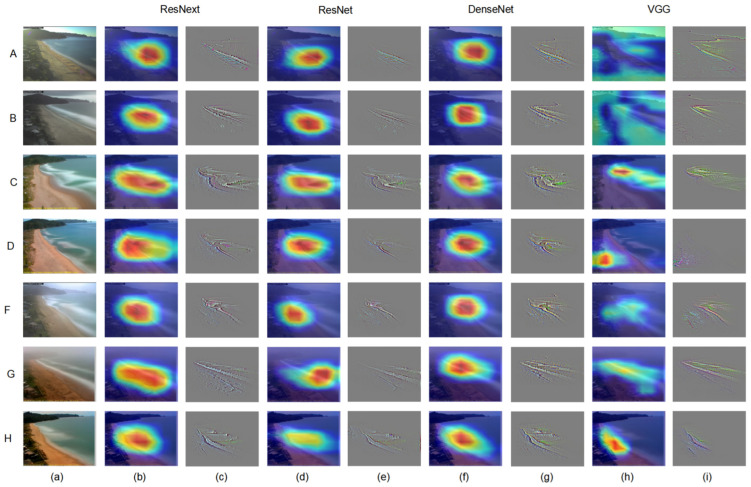
Comparison of visual results for different networks. (**a**) Original images for classes A, B, C, D, F, G, H. (**b**,**d**,**f**,**h**) Grad-CAM: locates category-distinguishing regions according to various visualizations for ResNext, ResNet, DenseNet, and VGG. Brighter colors in the graph represent higher category scores. (**c**,**e**,**g**,**i**) Guided Grad-CAM: high-resolution visualization of texture information used for category differentiation. Note that all these visualizations are based on features from the last CNN layers (before the fully connected layer).

Class Activation Map (CAM) uses feature visualization to explore the working mechanism and judgment basis of deep convolutional neural networks, which is widely used in the field of deep learning interpretability. In this section, we built attention maps using Grad-CAM and Guided Grad-CAM [52] as tools to provide visual analysis between the proposed deep learning architectures and others (shown in Figure 5).

Convolutional neural networks such as ResNet, VGG, and DenseNet have repeatedly stacked the multiple convolution layer and pooling layer whose last layer contains the most abundant spatial and semantic information which is difficult for humans to understand. Therefore, in order for the convolutional neural network to give a reasonable explanation of its classification results, it is necessary to make full use of the last convolutional layer. Grad-CAM is class-discriminative and localizes relevant image regions. Specifically, it can use the gradient of any target following the selected layer (last convolutional layer) in the CNN model to generate a heat map that highlights the important region in the image for predicting the castigator. The maps also have an associated score, indicating the probability of each label, which determines the final classifier response. Guided Grad-CAM fuses Guided Backpropagation and Grad-CAM visualizations to highlight fine grained details (textures and shapes) of images that determine what areas the neural network pays more attention to.

Figure 5 shows different attention maps of ResNext, ResNet, DenseNet, and VGG, using Grad-Cam and Guided Grad-CAM tools. Overall, (b, d, f, h) show that the neural network makes classification decisions mainly based on beaches and nearshore marine areas, and the regions with the highest scores (red in the picture) are mainly concentrated near the coastline. Additionally, the contribution to the classifier gradually decreases with increasing distance from the shoreline. Further, (c, e, g, i) clearly show that the gradient information on which the neural network updates the network parameters is mainly contributed by the beach and wave morphology. Specifically, the Grad-CAM of (b, d, f) is relatively homogeneous and concentrated, and the highlighted area is concentrated along the coastline, indicating that the network positioning is relatively accurate. However, in terms of the extent and trend of the activation regions, ResNext performs best, followed by DenseNet and ResNet. (h) columns show unfitted phenomena (A, B) and incorrect activation region (D), leading to the wrong prediction of the model, and thus VGG performs worst. In addition, it can be seen from Guided Grad-CAM (c, e, g, i) that ResNet and DenseNet retain beach and wave morphology relatively intact in terms of detail which determines the category of the image, while ResNet is under-detailed and VGG contains useless background information (A, B, F) or incorrectly located gradients (D). It may be due to the excessively complex network structure and large number of parameters of VGG that training is difficult.

In summary, the visualization techniques based on Guided Grad-CAM and Grad-CAM help us to understand intuitively how CNN predict beach categories. Meanwhile, we find that ResNext can make better use of beach and wave morphological texture information to obtain the best prediction results. In addition, it should be pointed out that the CNN model could maintain a high accuracy only by simultaneously detecting the morphology of the beach and wave.

### 4.2. Experiments on Motif Discovery

In the above experiments, we achieved good results in the classification task of a dataset, so that we tried to use motif discovery algorithm to further study state transition. Sequential pattern mining algorithms (FP-Growth [53] and Prefixspan [54]) were considered for comparison.

According to FP-Growth, Prefixspan, and MDLats algorithms, we need to generate a sequence dataset. First, we used the best model obtained from the above experiment to predict the category of the unlabeled images from 1999 to 2008. Then, we generated two sequential data by partitioning the forecast results by time. The sequential dataset is divided by day. The category names A, B, C, D, F, G, H are represented by 0, 1, 2, 3, 4, 5, 6, respectively.

In Table 5, the results of three algorithms show similar trend behaviors. We find that the frequency of occurrence of states 0, 2, and 4 is relatively high, and the patterns or frequent items containing two or more of these states are observed. The other states 1, 3, 5, and 6 are the least observed and almost never form part of a motif pattern. This is also confirmed in Figure 7. Moreover, comparing the left column with the middle column, since the input data of FP-Growth algorithm are the collection representation, it only shows which states frequently occur and does not describe any pattern. The Prefixspan and MDLats algorithms can instead find frequent sequences. Comparing the middle column with the right column of Table 5, both algorithms can find trivial no-change patterns such as [0, 0, 0, 0, 0, 0, 0, 0] and [2, 2, 2, 2, 2, 2, 2, 2, 2]. However, MDLats can generate all the motifs, including not only the most similar pairs of subsequences as commonly done in existing methods but also all the similar subsequences, which can find more patterns than Prefixspan algorithm and provide more information for time series analysis. For example, the states 3 and 5 both have a lower occurrence frequency, which cannot be found by Prefixspan but appearance in the motif sequence by MDLats. Thus, MDLats is more accurate and more suitable for our dataset than the other two methods.

In addition, the results only show short-term (within one day) patterns, the longest of which is no more than 14 (the largest number of images collected in a single day). The reason is the lack of night data, which resulted in discontinuous beach state, and thus the state sequence could only be divided by day. If this effect is ignored, a longer pattern may be found, which we left for future work.

## 5. Discussion

**Figure 6:**
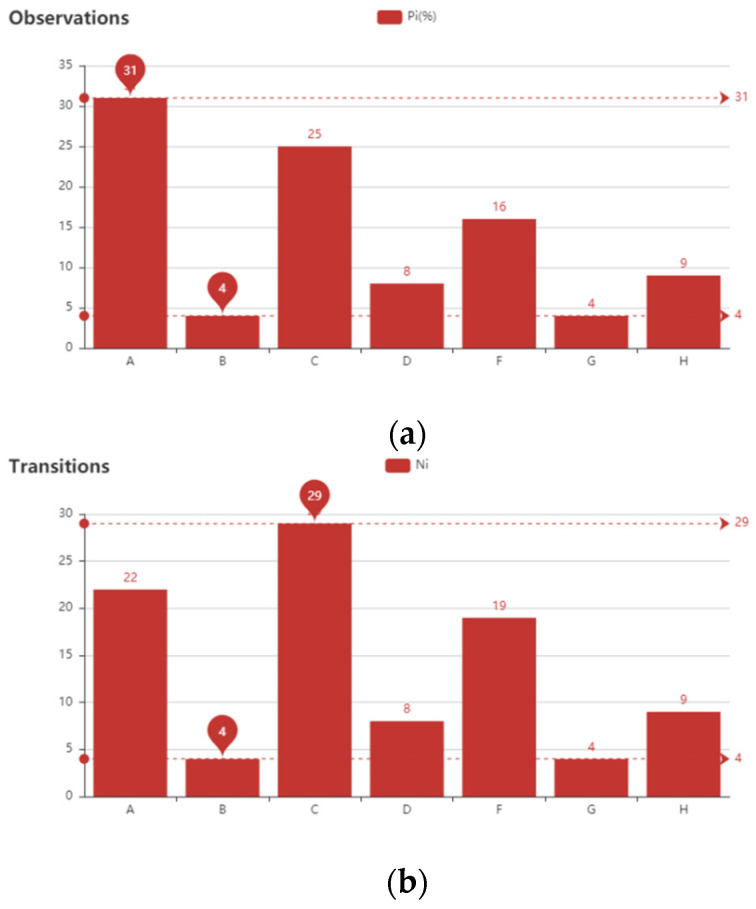

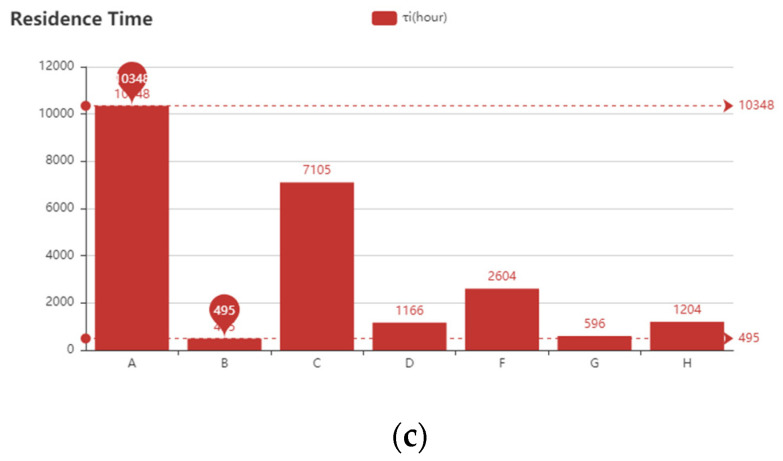
Three statistical indicators of all beach types. (**a**) Occurrence percent of beach type (%) Pi, which is computed by the number of days of one bar type observed dividing the total number of sample days. (**b**) Transition of beach type (%) Ni, which is calculated by the number of one beach state transforming to other states dividing the total number of transitions. (**c**) Residence time of bar type (hours) τi.

We carried out statistical analysis on the results (as shown in Figure 6). The data in the table are based on daily measurements from 1999 to 2008.

With respect to beach transition and residence time, different states have almost the same tendency. As shown in Figure 6, the most common state observed is reflective bar, bar type A (Pi = 31%). Although bar type C (21%) ranks second, it occurs on many different occasions as bar type changed (NH = 29%). Bar types B and G, which occur with almost the same percentage, are seen on progressively fewer occasions. Bar types B, D, and G all occur less than 10% of the time. The results are consistent with actual observations which have been made to once again verify the accuracy of our model predictions.

In addition, incident scaled features (Type B) are never observed in bars with longshore variability and only occurred in the form of quasi-straight ridge-and-runnel or low-tide terrace bars. The differences in Ni and Pi are attributed to the relative stability of the individual morphologic forms [3].

**Figure 7:**
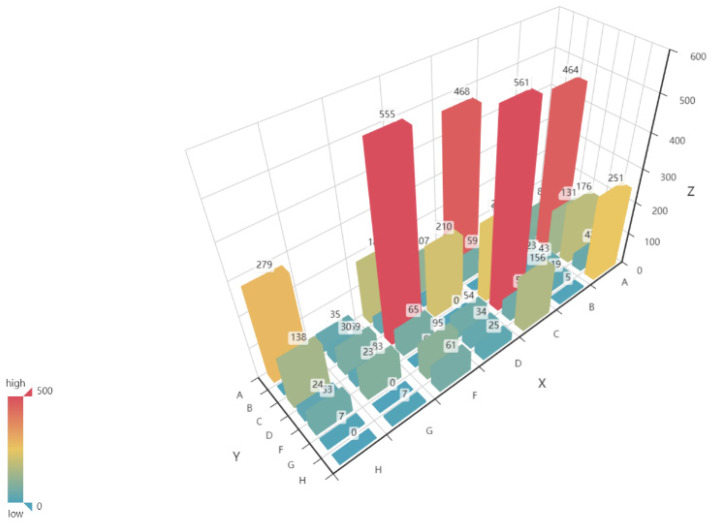
The number of transitions between morphologic bar types. The X coordinate represents the initial type, Y represents the following type, (x, y) represents the transition from type x to type y, and Z represents the number of type transitions.

Furthermore, as shown in Figure 7, we studied the transition between the various states. Transitions to lower bar types (above the diagonal) are unusual, with 19.6% of downward transitions between neighboring states. Transitions to higher bar types (below the diagonal) are similar (21.1%). The transition type C → F and F → C is the most common (21.6% as an upward transition, 21.8% as a downward transition) followed by the transition A → C and C → A (17.9%, 18.6%) and A → H and H → A (9.7%, 11.1%). Thus, it is unusual to have a low proportion of transitions between successive states and a high proportion of transitions across multiple states. However, this result may be a natural consequence of the time scale response of the sandbar to the wave forcing. Interestingly, the number of transitions between each two bar types is approximately the same and the proportion of upward transitions is higher than that of downward transitions.

**Figure 8:**
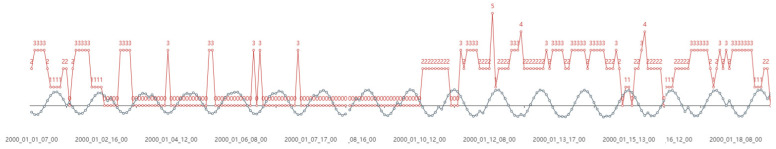
The hourly type and tidal height from 7 January 2000 to 18 January 2000. The red line indicates the bar type at each hour, while the blue line represents the height of tide.

Figure 8 shows the hourly bar type and tidal height from 7 January 2000 to 18 January 2000. An average of 12 images was available each day. Initially, as a result of wave breaking over the bar being more pronounced or absent depending on water levels, the beach state changed regularly between low and high tides. Later, when wave conditions increased, such pattern is lost and the beach experiences more energetic states, the tidal effect is still present but less evident.

We must point out that beach state changes have certain limitations in time and space. As we described in Section 3.1.1, each beach image represents a beach state which is defined by discrete morphologic descriptions. However, sandbars show a continuum of shapes as they evolve between configurations [32]. Therefore, there may be multiple states or complex states in a given image, that may lead to the difficulty in determining what type of beach state a particular image belongs to. In the process of image annotation by experts, perception differences inevitably exist. In addition, the lack of nighttime data makes the transform of beach state not smooth. Moreover, the performance of the deep learning model is closely related to the dataset scale of training, which is also an important reason limiting the application of our method.

In addition, we compared our method with the latest work which is similar to ours. Ellenson et al. [39] verified the accuracy and transfer ability of their CNN (ResNet) model based on two data sets from Narrabeen (New South Wales, Australia) and Duck (North Carolina, USA). They find that when the model trained on a single beach dataset is used to predict a new beach dataset, its performance is seriously degraded. The effect of the model trained by multiple beach datasets is slightly worse than that of the model trained by a single beach, but its generalization ability is actually better. In this paper, we improved the prediction accuracy and generalization ability of a single model (ResNext) by self-training mechanism in the case of a single large dataset with few labeled data. However, we did not verify our method for other beaches, and we will conduct further experiments on other beaches in the future. We also compared our method with traditional methods based on image processing and analyzing. Armaroli et al. [32] used an Argus automatic tool to get the shapes of bars and breaking waves to classify the beach. However, they ignored location of the longshore and cross-shore of the bar crest and the relationship between the outer and the inner bar or with the shoreline. In this paper, CNN model can integrate all above features in a nonlinear fitting way to determine the final beach category. Moreover, Argus procedure is time-consuming and the time spent following the procedure increases together with the complexity of the study site, while the CNN model identifies beach type in milliseconds and can be applied to any site as long as there are enough images for training.

## 6. Conclusions

In this paper, a beach classification method based on deep learning was proved to be effective. By comparing multiple CNN models, ResNext obtained the best accuracy of 0.9365. Meanwhile, we integrated a self-training mechanism based on the model to obtain the best classification accuracy (*F1*-score = 0.9014). Further, visualization techniques (Guided Grad-CAM and Grad-CAM) were used to identify specific image areas for CNN decision-making. The results show that CNN focuses on the area of interest related to beach and near-shore wave, and obtains the location and shape information of offshore dam, coastline and wave of coastal dam, and trough, which is finally used for classification decision.

A pattern recognition method was proposed to identify beach state transitions according to the classification results. After grouping and serializing the data of beach state over the past 20 years, the frequent beach patterns could be recognized every year by using a data mining algorithm to study the change of beach state. At the same time, combined with the corresponding monitoring data and statistical analysis of prediction results, we find that the most common state of Tairua Beach observed is reflective bar. The bar presents as states C and D at low tide and state F at high tide. The proportion of transitions between continuous states is low, while the proportion of transitions across multiple states is high.

In the future, natural extensions of this work imply developing a predictor of beach states that accounts for wave conditions and also attempting to automatically identify rip current occurrence.

## Figures and Tables

**Table 1:** Ablation results of image enhancement results (bold means the best result).

Data Processing	Algorithm	Top-1 Accuracy
No image enhancement	ResNet50	0.9028
	ResNext50	0.9146
Image enhancement	ResNet50	0.9278
	ResNext50	**0.9365**

**Table 2:** Ablation results of self-training algorithm (bold means the best result).

	A	B	C	D	F	G	H	Avg
ResNext50	0.9470	0.7960	0.9462	**0.9343**	0.8881	0.9550	0.8435	0.9014
ResNext50 + ST	**0.9547**	**0.8479**	**0.9572**	0.9149	**0.9085**	**0.9883**	**0.8518**	**0.9176**

**Table 3:** *F1*-score with different classifications networks (bold means the best result).

	A	B	C	D	F	G	H	Avg
ResNet50 + ST	0.9487	0.7891	0.9475	**0.9182**	0.8790	0.9550	0.8095	0.8924
ResNext50 + ST	**0.9547**	**0.8479**	**0.9572**	0.9149	**0.9085**	**0.9883**	**0.8518**	**0.9176**

**Table 4:** Comparison of prediction performance with different models (bold means the best result).

Method	Precision	Recall	*F1* Score	Referring Time (ms)
VGG16	0.8754	0.8875	0.8858	50.0
DesNet121	**0.8960**	0.8870	0.8890	35.6
ResNet50	0.8870	0.9014	0.8924	**17.3**
ResNext50	0.8714	**0.9308**	**0.9014**	18.4

**Table 5:** Persistency patterns and motifs discovered by FP-Growth (left), Prefixspan (middle), and MDLats (right). Each row of data on the left represents frequent item, in the middle represents frequent sequence, and on the right represents motif. The occurrence frequency of each pattern or motif in the figure gradually decreased from top to bottom.

FP-Growth	Prefixspan	MDLats
{0}	[0, 0, 0, 0, 0, 0, 0, 0]	[0, 0, 0, 0, 0, 0]
{2}	[0, 0, 0, 0, 0, 0, 0, 0, 0]	[0, 0, 0, 0, 0, 0, 0]
{2, 0}	[2, 2, 2, 2, 2]	[0, 0, 0, 0, 0, 0, 0, 0]
{4}	[0, 0, 0, 0, 0, 0, 0, 0, 0, 0, 0,0]	[0, 0, 0, 0, 0, 0, 0, 0, 0]
{2, 4}	[2, 2, 2, 2, 2, 2, 2, 2, 2, 2]	[2, 2, 2, 2, 2, 2, 2, 2, 2]
{4, 0}	[2, 2, 2, 2, 2, 2, 2, 2, 2, 2, 2]	[0, 0, 0, 0, 0, 0, 0, 0, 0, 0, 0, 0]
{6}	[0, 0, 0, 0, 0, 0, 2]	[2, 2, 2, 2, 2, 2, 2, 2, 2, 2, 2]
{1}	[0, 0, 0, 0, 0, 2, 2]	[4, 4, 4, 4, 4, 4, 4, 4]
{0, 6}	[0, 0, 0, 6]	[2, 0, 0, 0, 0, 0]
{2, 4, 0}	[2, 2, 2, 2, 2, 2, 2, 2, 0]	[0, 0, 0, 0, 0, 2]
{1, 2}	[4, 4, 4, 4, 4, 4, 4]	[4, 0, 0, 0, 0, 0, 0, 0]
{1, 0}	[4, 0, 0, 0, 0]	[0, 0, 0, 0, 0, 0, 6]
{2, 6}	[0, 0, 0, 0, 0, 0, 2, 0]	[4, 4, 4, 4, 4, 4, 4, 4, 4, 4]
{2, 0, 6}	[6, 0, 0, 0, 0, 0, 0]	[6, 0, 0, 0, 0, 0, 0]
{1, 2, 0}	[0, 6, 0, 0, 0, 0, 0]	[0, 0, 0, 0, 0, 2, 2]
{1, 4}	[0, 0, 0, 0, 0, 6]	[5, 5, 5, 5, 5, 5]
{5}	[0, 0, 0, 2, 2, 2, 0]	[0, 0, 0, 2, 2, 2]
{3}	[4, 2, 2, 2, 2, 2]	[0, 0, 0, 6, 6, 6]
{4, 6}	[2, 2, 2, 2, 2, 2, 2, 2, 2, 0]	[2, 2, 2, 2, 2, 0]
{1, 2, 4}	[0, 0, 2, 2, 2, 2, 2, 2, 2]	[0, 0, 0, 0, 0, 6]
{1, 4, 0}	[2, 2, 2, 4, 2, 2]	[6, 0, 0, 0, 0, 0, 0, 0, 0]
{4, 6, 0}	[6, 0, 0, 0, 0, 0, 0, 0]	[3, 3, 3, 3, 3, 3]
{5, 0}	[0, 0, 0, 0, 0, 0, 4]	[1, 1, 1, 1, 1, 1, 1, 1, 1]
{2, 3}	[2, 2, 2, 6]	[2, 2, 2, 0, 0, 0, 0]
{0, 3}	[0, 6, 6, 0, 0, 0]	[6, 6, 6, 6, 6, 6]
{2, 4, 6}	[6, 6, 6, 6, 6]	[5, 5, 5, 5, 5, 5, 5]
{5, 2}	[0, 1, 2, 2]	[2, 2, 2, 2, 2, 3]

## Data Availability

We have no intention to open source data for the time being.
